# Identification of a gene signature of a pre-transformation process by senescence evasion in normal human epidermal keratinocytes

**DOI:** 10.1186/1476-4598-13-151

**Published:** 2014-06-14

**Authors:** Nathalie Martin, Clara Salazar-Cardozo, Chantal Vercamer, Louise Ott, Guillemette Marot, Predrag Slijepcevic, Corinne Abbadie, Olivier Pluquet

**Affiliations:** 1 CNRS, UMR8161, Institut de Biologie de Lille, 1 rue Calmette, 59000 Lille, France; 2 Université Lille 1 Sciences et Techniques, 59650 Villeneuve d’Ascq, France; 3 Université Lille 2 Droit et Santé, 59000 Lille, France; 4 Institut Pasteur de Lille, 59000 Lille, France; 5 Inria Lille-Nord Europe MODAL, 40 avenue Halley, 59650 Villeneuve d’Ascq, France; 6 EA2694 Université Lille 2 Droit et Santé, 59000 Lille, France; 7 Brunel Institute of Cancer Genetics and Pharmacogenomics, Brunel University, Uxbridge, UK; 8 INSERM UMR_S 1109, Centre de Recherche d’Immunologie et d’Hématologie, Fédération de Médecine Translationnelle de Strasbourg (FMTS), Université de Strasbourg, Strasbourg Cedex, France

**Keywords:** Senescence, Neoplastic transformation, Xenobiotics, AKR1Cs, Keratinocytes, Gene expression profile

## Abstract

**Background:**

Epidemiological data show that the incidence of carcinomas in humans is highly dependent on age. However, the initial steps of the age-related molecular oncogenic processes by which the switch towards the neoplastic state occurs remain poorly understood, mostly due to the absence of powerful models. In a previous study, we showed that normal human epidermal keratinocytes (NHEKs) spontaneously and systematically escape from senescence to give rise to pre-neoplastic emerging cells.

**Methods:**

Here, this model was used to analyze the gene expression profile associated with the early steps of age-related cell transformation. We compared the gene expression profiles of growing or senescent NHEKs to post-senescent emerging cells. Data analyses were performed by using the linear modeling features of the limma package, resulting in a two-sided *t* test or F-test based on moderated statistics. The p-values were adjusted for multiple testing by controlling the false discovery rate according to Benjamini Hochberg method.

The common gene set resulting of differential gene expression profiles from these two comparisons revealed a post-senescence neoplastic emergence (PSNE) gene signature of 286 genes.

**Results:**

About half of these genes were already reported as involved in cancer or premalignant skin diseases. However, bioinformatics analyses did not highlight inside this signature canonical cancer pathways but metabolic pathways, including in first line the metabolism of xenobiotics by cytochrome P450. In order to validate the relevance of this signature as a signature of pretransformation by senescence evasion, we invalidated two components of the metabolism of xenobiotics by cytochrome P450, AKR1C2 and AKR1C3. When performed at the beginning of the senescence plateau, this invalidation did not alter the senescent state itself but significantly decreased the frequency of PSNE. Conversely, overexpression of AKR1C2 but not AKR1C3 increased the frequency of PSNE.

**Conclusions:**

To our knowledge, this study is the first to identify reprogrammation of metabolic pathways in normal keratinocytes as a potential determinant of the switch from senescence to pre-transformation.

## Background

The progression from normal cells to cancer is a multistep process that requires a wide variety of molecular alterations. To override the normal mechanisms controlling cellular proliferation, transformed cells accumulate somatic or inherited mutations in several tumor suppressor genes, oncogenes and genes that are involved in maintaining genomic stability [1,2]. Another driving condition of transformation is the chromosomal instability which includes loss of heterozygosity (LOH), aneuploidy, chromosome translocation and gene amplifications [3]. In addition, epigenetic silencing through DNA methylation has been shown to strongly affect the activity of tumor suppressor genes and oncogenes during malignant transformation [4,5]. Therefore, the phenotype and natural history of transformed cells is determined by the accumulation of changes and by the order of events. In all cases, cells are fully transformed to the neoplastic stage only when they acquired functional capabilities to survive, proliferate and disseminate despite genomic instability [6]. It is not yet clear which immediate pathways program preneoplastic lesions which then progress to a fully transformed phenotype. During the last decade, clinical, epidemiological, animal and gene expression profiling studies have been used to identify differentially regulated genes or pathways important for tumorigenesis. However, when transcriptional profiling utilizes cell lines, it often involved isogenically matched non-transformed and transformed cells through overexpression of telomerase (hTERT) and oncogenes such as v-Src [7], H-Ras [8]. These approaches make not possible to kinetically follow initial and intermediate stages between normal and fully transformed cells. Therefore, the current clinical models lack initial phases and the current *in vitro* culture models lack the importance of the order of the events.

Aging is associated with a number of events at the molecular, cellular and physiological levels that influence malignant transformation. It appears that the incidence of carcinomas, the most frequent type of cancer in humans, highly increases with age, unlike to other types of cancer (NCI cancer statistics). Effects of aging on the body are accompanied by an increase in senescent cells, which are viable but have lost their ability to divide [9]. Through its capacity to inhibit cell proliferation, senescence was recognized as a tumor suppressor mechanism [9]. However, several lines of evidence indicate that it would also have a tumor promoter role through non-cell and cell autonomous mechanisms. The senescent fibroblasts secrete inflammatory factors that can promote the proliferation of cancerous cells and the appearance of tumor clones [10]. In addition, we and others found that normal human mammary epithelial cells (HMECs) and normal human epidermal keratinocytes (NHEKs) can spontaneously escape from the senescent state, emerge to re-enter the cell cycle and undergo cell divisions [11,12]. In the case of NHEKs, we named this process post-senescence neoplastic emergence (PSNE). We demonstrated that clones of small cells emerge from senescent cells at a frequency between 10^−2^ to 10^−4^ by atypic mitosis but did not come from a pre-existing subpopulation. Emergent cells showed consistent cellular morphologies similar to normal growing cells compared to enlarged senescent cells associated with a loss of senescence-associated β-Gal activity. A Subcutaneous injection of emerging cells in nude mice induced after several months disseminated skin lesions (hyperplasia/small carcinomas), strongly suggesting the tumorigenic potential of emerging cells [12]. Characterization of emerging cells showed that they displayed normal karyotype [12], did not carry Ras mutation, did not form colony in soft agar [12] but can become invasive in an inflammatory environment [13]. This indicated that in culture the post-senescent emergence gave rise to pre-transformed cells with tumorigenic potential rather than cells at the fully-transformed neoplastic stage. Therefore, post-senescence emergence of NHEKs may represent an early stage in the process of transformation, mimicking a physiological mode of oncogenic transition in a context of aging.

In order to gain insights into the molecular basis of early transformation process, by using microarray transcriptomic data combined to bioinformatic analyses, we identified a specific gene expression signature of this pre-transformed phenotype. This signature comprises in particular genes involved in xenobiotics metabolism including the NADP (H)-dependent oxidoreductases AKR1C2 and AKR1C3. Further functional approaches confirmed the involvement of this new pathway in early steps of cancer initiation in a context of aging.

## Results

### Identification of a post-senescence neoplastic emergence (PSNE) gene expression signature

**Figure 1:**
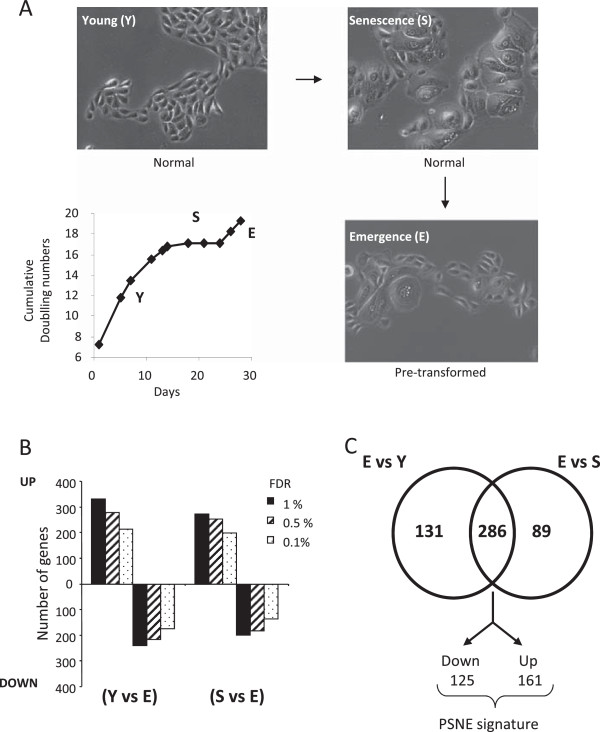
**Acquisition of the PSNE gene signature. (A)** NHEK growth curve and representative photomicrographs of senescent (S) and emergent NHEKs (E) as compared to NHEKs in exponential growth phase (Y) observed by phase-contrast microscopy. **(B)** Number of differentially up- and down-regulated genes in E versus Y NHEKs and E versus S NHEKs analysed with a range of FDR filters from 1 to 0.1%. **(C)** Venn diagram showing the PSNE gene signature defined by the overlap of differentially expressed genes in E vs Y and E vs S NHEKs.

**Figure 2:**
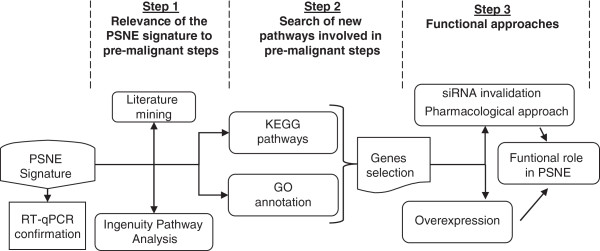
**Data mining strategy.** Shown is a schematic diagram outlining our genomic bioinformatics analyses of the data generated by microarray analysis and the following functional studies using pharmacological and genetic tools.

To establish a gene signature specific of the earliest steps of tumorigenesis, we performed a transcriptomic analysis of post-senescent emergent NHEKs in comparison with senescent and young ones. We harvested RNAs from NHEK cultures at exponential growth phase (Y), senescence plateau (S) or post-senescence emergence (E) (Figure 1A), reverse transcribed the RNAs and then hybridized them to RNG-MRC human set 25K microarrays as described in the methods section. Comparison between E vs Y and E vs S were performed with three biological replicates (from the same donor) and using a dye-swap strategy to limit color bias. The R package limma was used to test for differential expression. Variances were modelled using an empirical Bayesian approach in order to increase the sensitivity of the experiment and thus mitigate the low number of replicates. The visual observation of the MAPlots and the shape of p-values histograms (Additional file 1: Figure S1A and B) validate the statistical modelling, warranting further analyses and interpretation of differentially expressed genes. For delineating the differentially expressed genes, we applied as a first filter a range of False Discovery Rate (FDR) from 0.1 to 1% (Figure 1B). We retained the most stringent FDR value of 0.1%. As a second filter, we applied log (2) fold change (E/Y or E/S) ≥ 1.1. Hence, 417 genes differentially expressed between E vs Y and 375 genes between E vs S were selected. A Venn diagram analysis indicated that 286 genes were common to the two conditions. These 286 genes were all either up-regulated both in E vs Y and E vs S or down-regulated both in E vs Y and E vs S, suggesting that they represent the PSNE driver genes (Figure 1C). This gene set, hereafter called the PSNE signature, is presented in Additional file 2: Table S1. Validation of the array data was carried out on a subset of up- and down-regulated genes by quantitative real-time PCR (Additional file 1: Figure S2), hence validating the overall signature. The PSNE signature was then the basis of bioinformatics analyses. The flow chart of these analyses is depicted in Figure 2.

### The PSNE signature is relevant of premalignant states or established epithelial cancers

**Figure 3:**
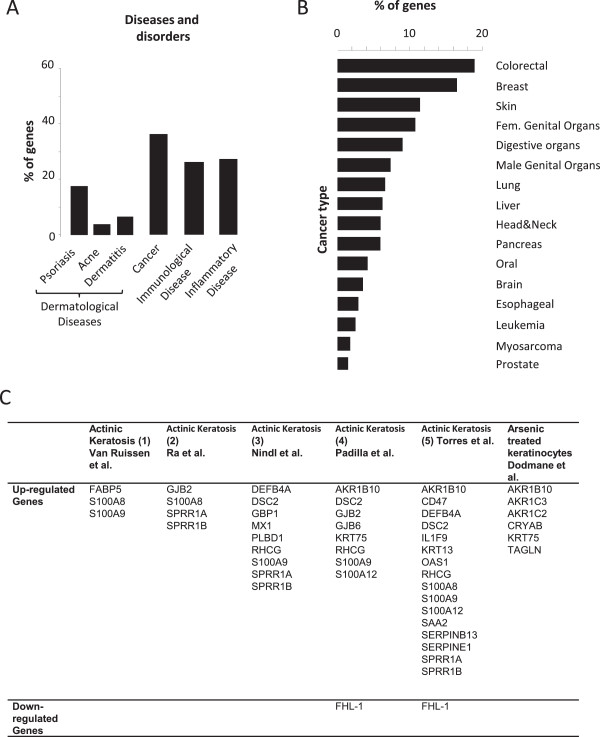
**Overlap between the PSNE gene signature and gene sets of cancer and premalignant related models. (A)** Ingenuity Pathway Analysis of the proportion of genes of the PSNE signature belonging to the Diseases and disorders category with a p value <0.0001. **(B)** Ingenuity Pathway Analysis of the percentage of genes of the cancer category in A involved in common cancer types. **(C)** List of the genes from the PSNE signature overlapping with data sets of pre-malignant non-melanoma skin pathologies from [14-19]. Were considered as overlapping only the genes differentially regulated in the same direction in each set.

In a first step (Figure 2), we wanted to evaluate whether the PSNE signature was relevant to cancerogenesis. To this end, we performed an analysis of the over-represented genes associated to diseases and disorders using Ingenuity software (Ingenuity Pathway Analysis (IPA), http://www.ingenuity.com). This analysis revealed that 36% of the PSNE genes are annotated in at least one cancer type (Figure 3A) and this was expanded to 50.7% (145/286 genes) by performing extensive literature mining. These 145 genes are highlighted in grey in Additional file 2: Table S1. It is worth to note that 18% of the PSNE genes are involved in psoriasis, a benign skin disease characterized by hyperproliferation of keratinocytes (Figure 3A), directly showing that PSNE can be used as a model for studying the initial steps of tumorigenesis. The comparison of the PSNE signature with those of common cancer types showed that the genes of the PSNE signature annotated in cancer (50.7%) are preferentially associated with the most frequent carcinomas, including colorectal, breast, skin and female genital organs carcinomas (Figure 3B), with a range of 4–54 genes (1.4-18.9%) within each cancer type category (Figure 3B). Finally, to determine whether the PSNE signature is relevant of early steps of carcinogenesis, and especially of early steps of non-melanoma skin carcinogenesis (NMSC), we compared by literature mining the PSNE signature with array-based transcriptional profiles of actinic keratosis (AK) and arsenic-induced cutaneous hyperplastic lesions, two premalignant state related models of NMSCs [14-19]. The PSNE gene set overlaps with a total of 28 genes belonging to one or more profiles of the aforesaid premalignant models (Figure 3C).

These results suggest that the PSNE gene signature is relevant to epithelial human cancers and comprises genes required in pre-malignant states, thereby validating our experimental system of *in vitro* PSNE as a model of carcinoma initiation.

### The PSNE signature is enriched in genes belonging to a pathway of xenobiotic metabolism

In a second step, we searched for new pathways which could be involved in the pre-malignant steps. To this goal, the PSNE signature was analyzed according to over-represented Gene Ontology (GO) terms within the “molecular function” and “biological process” groups. The top five of this ontology analysis is listed in Table 1. The “molecular function” GO analysis revealed that the most over-represented GO category is the oxidoreductase activity category (GO: 0016491) in which almost all genes are up-regulated. The four other best statistically over-represented categories comprise much less genes than in the oxidoreductase activity category. These are the phosphoric diester hydrolase activity category (GO: 0008081), the transaminase activity category (GO: 0008483), the neutral amino-acid transporter activity category (GO: 0015175) and the unfolded protein binding category (GO: 0051082) (Table 1 and Additional file 2: Table S1). The “biological process” GO analysis emphasized numerous genes involved in response to stress, and to a less extent response to chemical stimulus, external stimulus, cellular ketone metabolism and epidermis development (Table 1). Next, the PSNE signature was analyzed by functional annotation using the Kyoto Encyclopedia of Genes and Genome (KEGG), a compendium of genes annotated and organized by signalling pathway [20]. The most significant functional identified pathway concerns the metabolism of xenobiotics by cytochrome P450 (Table 2). It includes AKR1C3, AKR1C2, GSTA4, ADH5, GSTP1, MGST2, ALDH3A1 that are all up-regulated in E vs Y and E vs S. Interestingly, all these genes belong to the GO oxidoreductase activity category (Table 1 and group 3 in Additional file 2: Table S1) and AKR1C2, AKR1C3 in particular, intersected with NMSC pre-malignant gene sets (Figure 3C). The second most significant identified pathway is that of aminoacyl-tRNA biosynthesis including the IARS, WARS, AARS, GARS, MARS genes that are all down-regulated in E vs Y and E vs S (Table 2). The other pathways identified within the top five KEGG pathways are Glycine, serine and threonine metabolism, Glycolysis/Gluconeogenesis and Prion diseases. The same kind of analysis was performed using IPA Canonical pathways. Three amongst the top five pathways that were highlighted by the analysis were the same as those highlighted by the KEGG analysis, namely with the best p value the Metabolism of xenobiotics by cytochrome p450 including ADH5, AKR1C2, AKR1C3, MGST2, GSTA4, CYP4B1, ALDH3A1 and GSTP1, in second Glycine, serine and threonine metabolism and in the forth position Aminoacyl-tRNA biosynthesis. The other two are Interferon signaling and Aldosterone signaling in epithelial cells (Table 3).

**Table 1:** Functional categorization of genes from the PSNE signature

***Category***	***Term***	***P***-***value***	***Count up***	**% *up***	***Count down***	**% *down***	***group***
**GO molecular function**							
GO:0008081	Phosphoric diester hydrolase activity	0.00207	5	1.74	2	0.69	1
GO:0051082	Unfolded protein binding	0.00225	5	1.74	3	1.04	2
GO:0016491	Oxidoreductase activity	0.00292	21	7.34	1	0.34	3
GO:0008483	Transaminase activity	0.0041	0	0	4	1.39	4
GO:0015175	Neutral amino acid transmembrane transporter activity	0.0042	0	0	4	1.39	5
**GO biological process**							
GO:0042180	Cellular ketone metabolic process	2.02E-8	11	3.78	20	6.87	6
GO:0009605	Response to external stimulus	1.16E-6	24	8.24	13	4.46	7
GO:0008544	Epidermis development	2.44E-6	12	4.12	3	1.03	8
GO:0006950	Response to stress	6.51E-6	31	10.65	22	7.56	9
GO:0042221	Response to chemical stimulus	1.47E-5	28	9.62	15	5.15	10

The top five Gene Ontology terms within the “Molecular function” and the “Biological process” subcategories and associated p-values are listed. The number and percentage of up- and down-regulated genes in each subcategory are indicated. A group number was attributed to each subcategory in order to spot the genes belonging to each subcategory in Additional file 2: Table S1.

**Table 2:** KEGG functional annotation of genes from the PSNE signature

***KEGG pathway***	***P***-***value***	***Genes***
Hsa00980 : Metabolism of xenobiotics by cytochrome P450	0.0011	AKR1C3, AKR1C2, GSTA4, ADH5, GSTP1, MGST2, ALDH3A1
Hsa00970 : Aminoacyl-tRNA biosynthesis	0.0087	IARS, WARS, AARS, GARS, MARS
Hsa00260 : Glycine, serine and threonine metabolism	0.0234	CTH, SHMT2, PSAT1, CBS
Hsa00010 : Glycolysis/Gluconeogenesis	0.0315	LDHA, ADH5, BPGM, PCK2, ALDH3A1
Hsa05020 : Prion diseases	0.0322	IL1B, HSPA5, PRNP, IL1A

The top five over-represented molecular pathways of the PSNE signature using KEGG pathway analysis.

**Table 3:** IPA annotation of genes from the PSNE signature

***Ingenuity canonical pathways***	***P***-***value***	***Genes***
Metabolism of xenobiotics by cytochrome P450	0.000126	ADH5, AKR1C2, AKR1C3, MGST2, GSTA4, CYP4B1, ALDH3A1, GSTP1
Glycine, Serine and Threonine Metabolism	0.00138	PSAT1, CBS, GARS, CTH, PLCH2, PLCL1, SHMT2
Interferon signaling	0.000257	IFIT1, OAS1, MX1, IFNGR1, PSMB8
Aminoacyl-tRNA biosynthesis	0.000295	WARS, GARS, AARS, MARS, IARS
Aldosterone signaling in epithelial cells	0.001175	HSPA8, CRYAB, DNAJB4, HSPA9, PLCH2, DNAJA1, HSPA5, PLCL1, HSPB1

The top five over-represented molecular pathways of the PSNE signature using Ingenuity Pathway Analysis.

Taken together, these results suggest that genes encoding proteins carrying oxidoreductase activity and involved in the metabolism of xenobiotics may be crucial for the early steps of transformation in the PSNE cellular model.

### The oxidoreductase genes AKR1C2 and AKR1C3 promote PSNE

**Figure 4:**
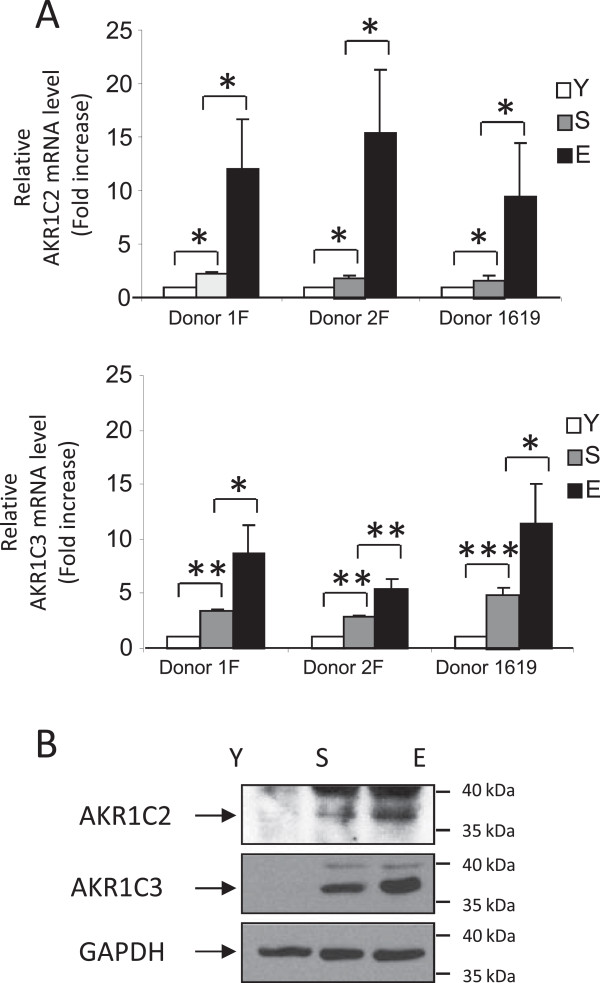
**AKR1C2 and 3 expressions in senescence and post**-**senescence emergence. (A)** The mRNA levels of AKR1C2 and 3 were measured by RT-qPCR in extracts from young (Y), senescent (S) and emergent (E) NHEKs obtained from three different donors. AKRC2 and 3 mRNA levels were normalized to 18S levels (Student t-test; *p ≤ 0.05; **p ≤ 0.01; ***p ≤ 0.001). **(B)** AKR1C2 and 3 protein levels were evaluated by Western Blot in extracts from young (Y), senescent (S) and emergent (E) NHEKs. The gel was equicharged with extracts from an equal number of cells. The equicharged was verified a posteriori by detecting the levels of GAPDH.

We then investigated by genetics and pharmacological approaches whether the AKR1C genes activated at senescence in primary keratinocytes may function as oncogenes, i.e. promote senescence evasion in the form of pre-transformed cells. We first verified by RT-qPCR the levels of AKR1C2, −C3 mRNAs in senescent NHEKs and emerging cells compared with young NHEKs. From RT-qPCR analyses performed with extracts from three different donors, we observed an increase in AKR1C2, −C3 levels at senescence followed by a much stronger increase at emergence (Figure 4A), hence confirming the microarray results. We next assessed whether the up-regulated transcription of AKR1C2, −C3 genes resulted in increased AKR1C2, −C3 protein levels. Western Blot analysis revealed strongly increased protein levels at senescence, further increased at emergence (Figure 4B).

**Figure 5:**
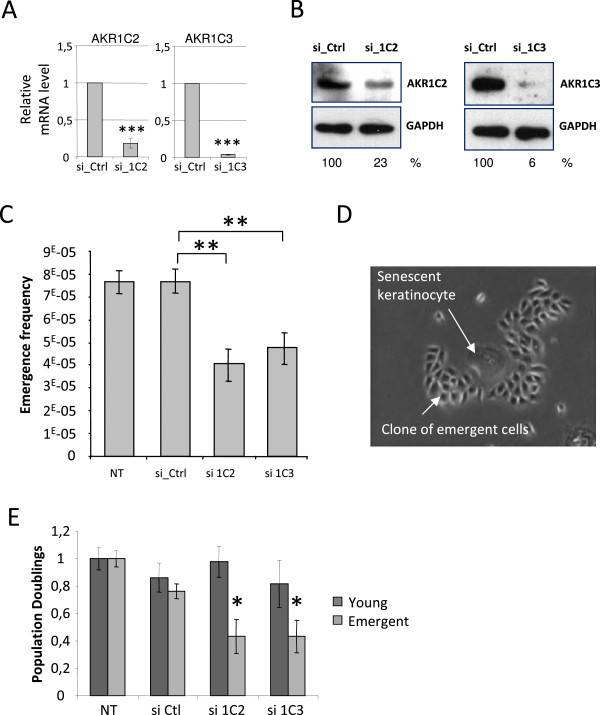
**Invalidation of AKR1C2 and 3 reduces preneoplastic transformation.** Senescent NHEKs were subjected to AKR1C2 (si_1C2) and AKR1C3 (si_1C3) silencing by siRNAs or to non-target siRNAs (si_Ctrl) for 4 days, or were not transfected (NT). **(A)** AKR1C2 and 3 mRNA levels were analyzed by RT-qPCR and normalized to 18S levels (Student t-test; ***p ≤ 0.001). **(B)** Protein extracts from an equal number of si_1C2, si_1C3 or si_Crtl NHEKs were analyzed by immunoblotting with anti-AKR1C2 or anti-AKR1C3 antibodies. Quantification of immunoblots was performed using Image J software, results are presented as the ratio AKR1C isoform/GAPDH. **(C)** The emergence frequency was determined by plating cells at low density and monitoring the culture for the appearance of emergent clones. Once emergent clones have appeared (after 7days), the culture dishes were stained by Crystal Violet and emergent clones were counted under microscopic observation. Countings were performed in triplicate (Student t-test; *p < 0.05; **p < 0.01, by comparing the conditions si_Ctrl with si_1C2 or si_1C3). **(D)** Emergent clone as exemplified by phase-contrast microphotograph. **(E)** Emergent or young cells were subjected to AKR1C2 (si_1C2) and AKR1C3 (si_1C3) silencing by siRNAs or to non-target siRNAs (si_Ctrl) during 4 days, or were not transfected (NT). Cells were counted and cumulative doubling numbers were calculated. The barplots represent the mean +/− s.d. of the counts of three independent culture dishes (Student t-test; *p < 0.05, of the comparisons of the conditions si_Ctrl with si_1C2 or si_1C3). This experiment is representative of 3 independent ones.

To establish whether AKR1C2 and –C3 play a role in PSNE, we examined whether decreasing their expression at senescence could affect the emergence frequency. To this goal, individual siRNAs directed against AKR1C2 or -C3 were transfected in senescent cells. As shown in Figure 5A and B, AKR1Cs siRNA transfection of senescent NHEKs resulted in strong decreased of AKR1C2, −C3 at both mRNA and protein levels. We first examined whether AKR1Cs siRNAs affected the senescent state itself. The results show that four days after transfection neither the population doubling numbers nor the percentage of SA-β-Gal positive cells were affected (Additional file 1: Figures S3A and S3B). We then monitored these siRNA-transfected senescent NHEKs for the emergence frequency. For that, siRNA-transfected senescent NHEKs were plated at low density, and, once the emergence has occurred about 7 days after transfection, the clones of emergent cells were manually counted and the emergence frequency calculated as the number of emergent clones on the number of initially plated senescent cells. The results show that knocking-down AKR1Cs diminished by a twofold factor the emergence frequency (Figure 5C and 5D). We also examined whether AKR1Cs could be involved in the proliferation of the emergent NHEKs. For that, emergent NHEKs were transfected with the siRNAs against AKR1C2 or –C3 or the control siRNAs and their proliferation was followed during 7 days. The results show that AKR1C2, −C3 knock-down inhibited proliferation of emerging cells from four days after transfection (Figure 5E). In contrast AKR1C2, −C3 silencing did not impact on cell proliferation in young NHEKs (Figure 5E). Taken together, these results indicate that AKR1Cs are involved both in the process of emergence from senescence and in the proliferation of the neo-formed transformed cells.

**Figure 6:**
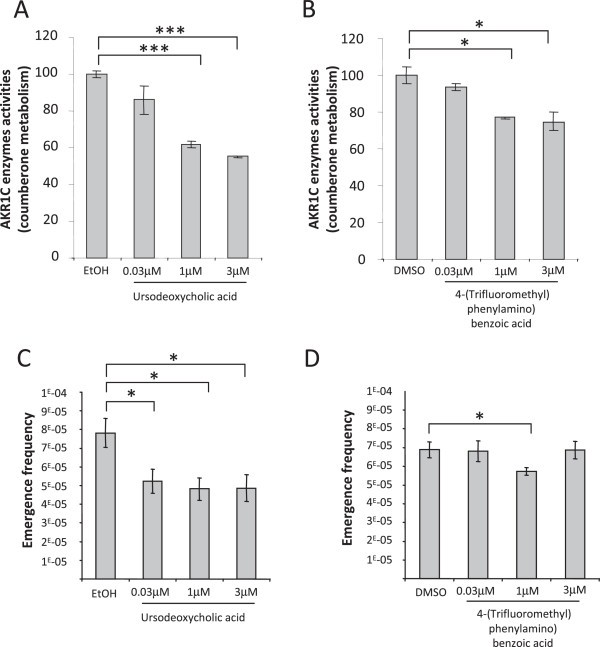
**Inhibiting AKR1C2 and 3 activities reduces preneoplastic transformation.** (**A** and **B**) Formation of fluorescent coumberol from non-fluorescent coumberone (AKR1Cs substrate) in 96-well plate of senescent keratinocytes in presence of increasing concentrations of AKR1C2 inhibitor (ursodeoxycholic acid) or AKR1C3 inhibitor (3-(4-(Trifluoromethyl) phenylamino) benzoic acid). Results are shown as fold induction +/− s.d. considering 100% the AKR1Cs activities obtained upon senescence. Each condition was performed in triplicate (Student t-test; *p ≤ 0.05; **p ≤ 0.01; ***p ≤ 0.001). The results are representative of at least 3 independent experiments. (**C** and **D**) The emergence frequency was determined by plating cells at low density and monitoring the culture for the appearance of emergent clones as in Figure 5D. Countings were performed in triplicate (Student t-test; *p < 0.05, of the comparison of the untreated condition (0 μM) with treated conditions (0.03; 1; 3 μM)).

**Figure 7:**
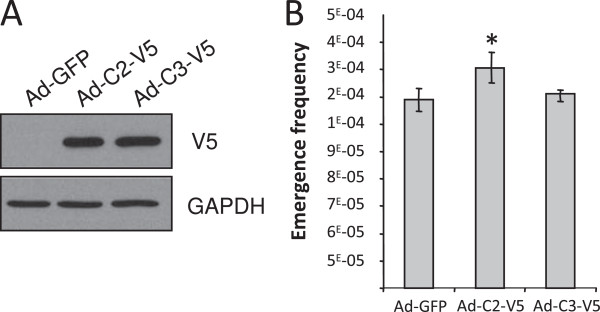
**Overexpression of AKR1C isoforms 2 and 3 promotes preneoplastic transformation. (A)** V5-tagged AKR1C isoforms 2 and 3 were overexpressed in senescent NHEKs from adenoviral vectors. Senescent NHEKs were infected with Ad-GFP, Ad-AKR1C2-V5 (Ad-C2-V5) or Ad-AKR1C3-V5 (Ad-C3-V5) particles, and analyzed 48 h later by western blot using an-anti V5 antibody and an anti-GAPDH used as loading control. **(B)** Once infected as in **(A)**, the emergence frequency was determined as in Figure 5D. Countings were performed in triplicate (Student t-test; *p < 0.05, of the comparison of the control-GFP condition with Ad-C2-V5/Ad-C3-V5 conditions).

We next wanted to determine whether AKR1C2, −C3 activity is required for post-senescence emergence. To this goal, we treated senescent NHEKs with the AKR1C2 inhibitor ursodeoxycholic acid [21] and AKR1C3 inhibitor 3-(4-(Trifluoromethyl) phenylamino) benzoic acid [22]. We first checked the efficacy of the inhibitors using coumberone, a non-fluorescent probe reporter, which is specifically metabolized by the AKR1C enzymes into fluorescent coumberol (see the methods section). We quantitatively measured coumberone metabolism in presence or absence of ursodeoxycholic acid or 3-(4-(Trifluoromethyl) phenylamino) benzoic acid in senescent NHEKs. We show that addition of both AKR1C inhibitors decreased coumberone metabolism, validating the inhibitory effects of the compounds (Figure 6A and B). We then determined the effects of AKR1C2, −C3 activity inhibition on senescence and emergence as above. Population doublings and emergence frequency were then determined as above. Both inhibitors did not affect senescence growth arrest, whatever the concentration used (Additional file 1: Figures S4A and B). Ursodeoxycholic acid significantly decreased the emergence frequency at the same rate whatever the dose (Figure 6C), while the AKR1C3 inhibitor was almost inefficient in impacting emergence (Figure 6D).To further support a role of AKR1C2, −C3 in pre-transformation, we investigated whether overexpressing AKR1C2, C3 in senescent NHEKs could also increase the emergence frequency. We used recombinant qdenovirus particles encoding V5-tagged AKR1C2, V5-tagged AKR1C3, or, as control, GFP. The overexpression of AKR1C2, −C3 were checked by western Blot using an anti-V5, 48h after infection (Figure 7A). We next followed the emergence frequency in AKR1C2, −C3 expressing cells. We showed that AKR1C2 but not AKR1C3 overexpression increased significantly the emergence frequency (Figure 7B).

In conclusion, these results indicate that the early steps of transformation in the PSNE model, including emergence from senescence and proliferation of the just emerged neoplastic cells are dependent, at least in part, on the expression and activity of AKR1C2 and to a less extent AKR1C3.

## Discussion

Although it is widely believed that there is a stepwise progression from normal cells to squamous cell carcinoma, the gene expression changes expected to induce the early oncogenic events that govern the early steps of malignant transformation are almost completely unknown. Since carcinomas always develop in a context of advanced age, we performed in this study transcriptomic and bioinformatics analyses to investigate the molecular events associated to the early steps of epithelial cell transformation by using a primary keratinocyte model progressing to senescence and giving rise to pre-neoplastic cells by a mechanism of senescence evasion.

This model offers several advantages and is relevant to the physiopathology of carcinogenesis for several reasons. First, it deals with primary epithelial human cells displaying a normal caryotype. Second, it enables to compare pre-transformed cells with the normal cells from which they directly originate, and not with neighbour normal cells either of another type or which were submitted to another environment as often the case when cells are microdissected off a tumor. Third, the comparison with normal cells can be done at two stages, when normal cells are still actively proliferating but also, and more interestingly, when they have reached the senescent stage. Since the pre-transformed cells directly originate from a few senescent cells [12], this enables to access to the transcriptomic changes which occur at senescence and prepare to transformation. In other words, the model can give access to a mechanistic understanding of the pre-transformation process by senescence evasion itself and not only to a description of the pre-transformed state once established. Forth, the pre-transformation process is completely spontaneous, i.e. it is not dependent on a priming experimental and sometimes artefactual event such as the introduction of a known oncogene, or even worse on unknown genetic modifications which have led to get an immortalized cell line. Therefore it opens the possibility to discover totally new pathways. And indeed, we found in the PSNE signature individual genes already annotated in cancer, in pre-neoplastic skin lesions or in psoriasis, but no global canonical pathway generally associated with cancer. For instance, in the IPA pathway analysis, the first canonical pathway generally associated with cancer that stands out is “HER-2 signaling in cancer”; it stands out only at the 15^th^ position. Similarly, in the KEGG analysis, the first canonical pathway relevant to cancer standing out is the “p53 signalling pathway”, only at the 8^th^ position.

An important consequence of the novelty of the model is our discovery in the PSNE signature of the preponderance of metabolic pathways. Amongst them, the “Metabolism of xenobiotics by cytochrome p450” is the one highlighted with the best p value and with the higher number of genes both by the IPA and KEGG analyses. Several of the genes of this pathway are also found in the oxidoreductase category of the IPA analysis. Remarkably, all these genes are up-regulated in the emergent cells versus the young or the senescent ones. Moreover, five on eight of these genes (AKR1C2, AKR1C3, GSTP1, MGST2 and ALDH3A1) are target genes of the oxidative stress-sensor NRF2 transcription factor. Since we have previously shown that oxidative stress is necessary and sufficient for both senescence and PSNE [12], this suggests that these genes might be amongst the actors of the process.

In order to assay this hypothesis, and more generally to prove that the PSNE model and the strategy to generate the PSNE signature has the potential to highlight pathways of importance in the process of pre-transformation by senescence evasion, we genetically and pharmacologically manipulated some genes of the “Metabolism of xenobiotics by cytochrome P450” and examined whether this could impact the process of emergence. Among the genes of the pathway, we choose to invalidate AKR1C2 and AKR1C3 because they were with CYP4B1 the most highly up-regulated and because they are known to be physiologically expressed in the epidermis [23-25]. AKR1Cs are a family of cytosolic NADP (H)-dependent oxidoreductases involved in the metabolism of steroids (C1-C3), prostaglandins (C3), polyaromatic hydrocarbons (C1-C3) and xenobiotics (C1, C2, C4,) [26-28]. Interestingly, the invalidation by RNA interference or the inhibition of AKR1Cs enzymatic activity by specific pharmacological inhibitors did not alter the senescent state itself, but significantly decreased the emergence frequency and the proliferation of the emerged cells. Conversely, AKR1C2 overexpression increased the frequency of PSNE, therefore validating the PSNE signature as a signature of the process of neoplastic emergence by senescence evasion in keratinocytes. However, in all genetic and pharmacologic manipulations of AKR1Cs, AKR1C2 revealed to be more efficient than AKR1C3 in promoting post-senescence evasion, probably because of differences in activity or substrate specificity. Therefore, although the overall PSNE signature can be considered as relevant of the very first stages of transformation, the exact implication of each gene must be experimentally assayed.

## Conclusions

Our results clearly indicate that the PSNE signature contains numerous informations which should be helpful in understanding basic molecular mechanisms involved in the very initial steps of pre-neoplastic transformation related to age. Further studies should focus on how the activity of the enzymes of the metabolism of xenobiotics by cytochrome P450 modifies the physiology of senescent keratinocytes to render them able to re-enter into mitosis to generate pre-transformed cells. Moreover, our findings suggest that a pharmacological intervention may prevent or delay early steps of neoplastic transformation. This may provide a foundation for future anti-aging and anti-cancer therapeutic approaches.

## Methods

### Reagents and cell culture

Normal human epidermal keratinocytes (NHEKs) were purchased from Clonetics (CC-2501) and Promocell. We used cells from 3 different donors of different race and age (referred as 4F0315, 2F1958, and K1MC). Cells were obtained anonymously and informed consent of each skin donor was obtained by the supplier. Cells were grown at 37°C in an atmosphere of 5% CO2 in a KGM-2 BulletKit medium consisting of modified MCBD153 with 0,15 mmol/L calcium, supplemented with bovine pituitary extract, epidermal growth factor, insulin, hydrocortisone, transferrin, and epinephrin (Clonetics). Such a serum-free low-calcium medium was shown to minimize keratinocyte terminal differentiation [29]. In all experiments, cells were seeded at 3500 cells/cm2 and always splitted at 70% confluence. The number of population doublings (PD) was calculated at each passage by means of the following equation: PD = log (number of collected cells/number of plated cells)/log2. Ursodeoxycholic acid and 3-(4-(Trifluoromethyl) phenylamino) benzoic acid were obtained from Sigma and Calbiochem, and diluted in ethanol and DMSO respectively.

### Emergence efficiency

After siRNA transfection, adenoviral infection or chemical treatment, senescent NHEKs were seeded into 10 cm dishes at the limit density for emergence (10,000 cells per dish). A few days later, the dishes were fixed and stained by crystal violet, the emergent clones were counted and the emergence frequency was calculated as the ratio of the number of clones on the number of seeded senescent cells [12].

### Transcriptomics analysis

#### cDNA preparation

Total RNAs were extracted using RNeasy mini-columns (QIAGEN). Double Stranded (DS) cDNA was synthesised from total RNA using a SMART protocol [30]. Two first strand reactions were set up starting with 500 ng of total RNA. Four microliters of each first strand reaction was used in the amplification step which was performed using 17 rounds of cycling. The DS cDNA was checked on a 1.2% agarose gel before the samples were purified using Qiagen Qiaquick clean up columns. The amount of cDNA amplified was then checked using the Nanodrop ND1000.

#### cDNA labelling and microarray hybridisation

Using the Bioprime labelling kits (Invitrogen), 10μl of purified cDNA was labelled, where 2 μl of Cye dye (GE) was incorporated. After incubating for 3 hrs at 37°C, the labels were purified using ProbeQuant G50 micro columns (GE). Incorporation rates were determined using a Nanodrop ND1000 before specific labels were pooled and dried down to completion. The labels were resuspended in 40 μl of hybridisation buffer (40% deionised formamide; 5× Denhart’s; 5× SSC; 1mM Na pyrophosphate; 50 mM Tris pH 7.4; 0.1% SDS) and hybridised onto a RNG-MRC human set 25K microarray printed on GE Codelink slide (http://www.mgu.har.mrc.ac.uk/facilities/microarray/rng.html), overnight at 48°C in a water bath using the Corning hybridisation chambers. After hybridisation, the arrays were then washed initially in 2 x SSC until the coverslip had come off, then 5 min with vigorous shaking in 0.1× SSC; 0.1% SDS and then finally in 0.1 x SSC for 2 min with vigorous shaking. The arrays were then spun dry and scanned using a ProScanArray HT (Perkin Elmer, Beaconsfield, UK) and the acquired images were analyzed using ImaGene 6.0.1 software (Bio Discovery, El Segundo, CA, USA). Three biological replicates were used for each of the young (Y), senescent (S), emergent (E) conditions. Dye-swap hybridizations were performed with the samples labelled reciprocally (Cy5 vs Cy3) to eliminate the influence of dye bias for a total of 9 arrays.

#### Analysis of microarray

Raw Data were processed into R/Bioconductor (http://www.bioconductor.org). Intensities were log2 transformed and normalized using the Loess method [31]. The fitted values for each sample were then converted back to red and green intensities. Moderated t-tests were performed using the limma package [32] to compare the following conditions: E vs Y and E vs S. Raw p-values were adjusted for multiple testing with the Benjamini Hochberg method. A false discovery rate (FDR) of 0.1% [33] and Log2 (Fold change) cutoff ≥ 1.1 was applied to the dataset for each condition [34]. We determined the common set of differentially expressed genes shared between E vs Y and E vs S as the PSNE signature. Expression data sets containing gene identifiers (Entrez gene identifiers) were uploaded and each gene identifier was mapped to its corresponding gene object in the Ingenuity Pathways Knowledge Base. The list of up- and down-regulated genes was analyzed using *Ingenuity Pathway Analysis* software 9.0 (http://www.ingenuity.com), and categorized according to their Gene Ontology (using DAVID, http://www.david.abcc.ncifcrf.gov/).

### RNA isolation, reverse transcription and quantitative real-time PCRs (qRT-PCR)

Total RNAs were extracted using RNeasy mini-columns (QIAGEN). One μg of RNA was reverse-transcribed using random hexamers, Superscript III and dNTPs (Invitrogen) in a final volume of 20 μl according to manufacturer’s instructions. Quantitative real-time PCR reactions were performed using the Mx3005P Real-time PCR system (Stratagen). Primers used were designed with qPrimerDepot (http://primerdepot.nci.nih.gov/) and are listed in Additional file 3: Table S2. PCR products were measured by SYBR Green fluorescence (SYBR Green Master Mix, Applied Biosystems). Experiments were performed in triplicates for each data point. Results were analyzed with the MxPro software (Agilent). The expression of each gene was normalized to 18S gene, and fold expression relative to the control is shown.

### Small interference RNA

Invalidation experiments for AKR1C2 and AKR1C3 were performed using pools of siRNAs from Dharmacon (ON TARGET Plus Smart pool, Dharmacon, USA). A non-targeting siRNA pool (Dharmacon) was used as control. Young, Senescent or Emergent NHEKs were plated at 70,000 cells per well in six-well plates and were transfected by using the RNAiMAX Lipofectamine reagent (Invitrogen Corp., Carlsbad, CA, USA) or by using PrimeFect siRNA Transfection Reagent (Lonza) according to manufacturer’s instructions.

### Western-blotting

Equal numbers of young, senescent or emergent NHEKs were lysed in Laemmli buffer 2× containing SDS 4%, Glycerol 20%, Tris–HCl 50 mM pH 6.8, bromophenol blue 0.02% and 2-Mercaptoethanol 5%. Samples were denatured by heating at 95°C for 5 min. Proteins were resolved by SDS-PAGE and transferred to nitrocellulose membranes (Hybond-C extra, Amersham). Primary antibodies used against AKR1C2 and AKR1C3 were from Abcam and anti-human GAPDH mouse monoclonal antibody was from Chemicon International, anti-V5 was from Life Technologies. Secondary antibodies used were peroxidase-conjugated (Jackson ImmunoResearch Laboratories). Peroxidase activity was revealed using ECL (enhanced chemiluminescence) or ECL advanced kit (Amersham Biosciences).

### Adenoviral infection

Adenoviral particles expressing AKR1C2-V5 (#SL170932) or AKR1C3-V5 (#SL177696) were purchased from SignaGen (Gaithersburg, MD, USA). AdGFP construction was previously described [35]. In all experiments, cells were infected at an input of 50 viral infectious particles/cells by adding virus stocks directly in the culture medium. Four hours later, the medium was replaced by fresh medium.

### AKR1C enzyme activity assay

The enzyme activity of AKR1C was assayed according to the procedure described by Halim et al. [36], with slight modifications. Briefly, cells were added to 96-well plates at a density of 6000 cells/well in phenol red-free media. Concentrations of inhibitors (as indicated) were added to the plate and incubated for 1 h, prior to addition of coumberone at concentration of 10 μM. Final volumes were 200 μl/well. The plates were returned to the incubator and fluorescence intensity was read 7 h after coumberone addition with excitation at 385 nm and emission at 510 nm on a FluoStar Optima platereader (BMG Labtech, Cary, NC). Results were expressed as a percentage +/− s.d. and normalized to control.

### Statistical analyses

Student’s one- or two-tailed *t* tests, as appropriate, were used for statistical analyses. The p values are indicated in the diagrams with * for p values < 0.05, ** for p values <0.01 or *** for p values <0.001.

### Ethics statement

Human cells used in this study provide from people whose informed consent was obtained by the cell suppliers (Clonetics, Promocell). Cells were obtained anonymously. No ethics approval was necessary for the experiments performed therein.

## Abbreviations

AKR1C: Aldo-keto reductase 1C; NHEK: Normal human epidermal keratinocyte; AK: Actinic keratosis; PSNE: Post-senescence neoplastic emergence; NMSC: Non-melanoma skin carcinogenesis.

## Competing interests

The authors declare that they have no competing interests.

## Authors’contributions

NM and OP conceived and designed the experiments. NM, CV, CSC and OP performed the molecular studies. PS carried out the microarrays. GM and LO performed the statistical analysis of microarrays. NM, CA and OP analyzed the data. OP and CA wrote the paper. All authors read and approved the final manuscript.

## Supplementary Material

Additional file 1: Figure S1 Quality assessment of the microarray data. (A) MAPlots of log-ratio of two expression intensities versus the mean log-expression of the two upon loess normalization in the different conditions (E vs Y; E vs S). (B) Histograms of raw p-values representing the number of p-values that falls within different intervals. **Figure S2.** Validation of the microarray data by quantitative real-time PCR. The mRNA level of a random subset of genes were measured by qRT-PCR and normalized to 18S levels. The barplots represent the mean +/- s.d. of three independent experiments. **Figure S3.** AKR1C2 and 3 expressions do not alter the senescent state. Senescent NHEKs were subjected to AKR1C2 (si_1C2) and AKR1C3 (si_1C3) silencing by siRNAs or to non-target siRNAs (si_Ctrl) for 4 days, or were not transfected (NT). (A) SA-b-Gal-positive cells were counted in 3 different microscopic fields. The barplots represent the mean +/- s.d. of the 3 counts. (B) Cells were counted and cumulative doubling numbers were calculated. The barplots represent the mean +/- s.d. of the counts of three independent culture dishes. **Figure S4.** AKR1Cs inhibitors do not alter senescence growth arrest. (A and B) Senescent NHEKs were treated with ursodeoxycholic acid or 3-(4-2 (Trifluoromethyl) phenylamino) benzoic acid. After four days, cells were counted and cumulative doubling numbers were calculated. The barplots represent the mean +/- s.d. of the counts of three independent culture dishes. Click here for file

Additional file 2: Table S1 The PSNE signature. List of the 286 genes of the PSNE signature. The 145 genes belonging to the cancer category in Figure 3A are highlighted in grey. The lane Group indicates the group numbers corresponding to the functional subcategories listed in Table 1 to which each gene belongs. Click here for file

Additional file 3: Table S2 List of primers for qRT-PCR. Click here for file
