# Supplementary figures and images for: Identification of a gene signature of a pre-transformation process by senescence evasion in normal human epidermal keratinocytes
Source: Mol Cancer. 2014 Jun 14;13:151. doi: 10.1186/1476-4598-13-151 (PMC4065601; doi:10.1186/1476-4598-13-151)

A

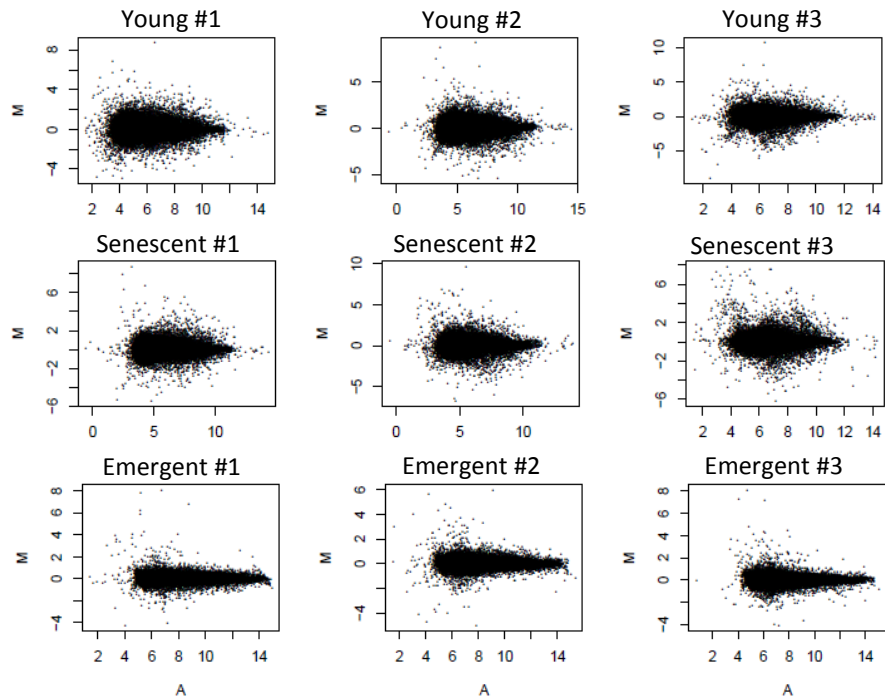

B

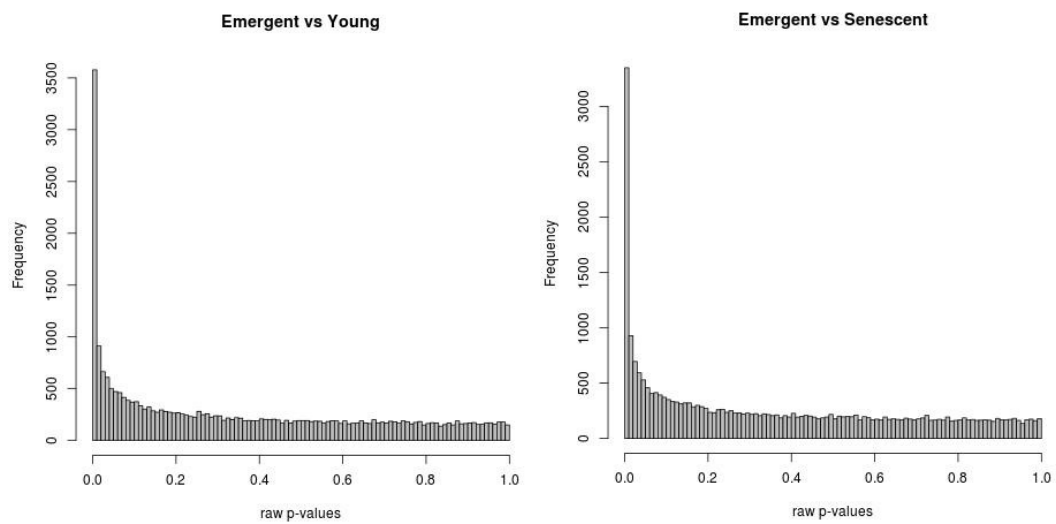

Figure S1

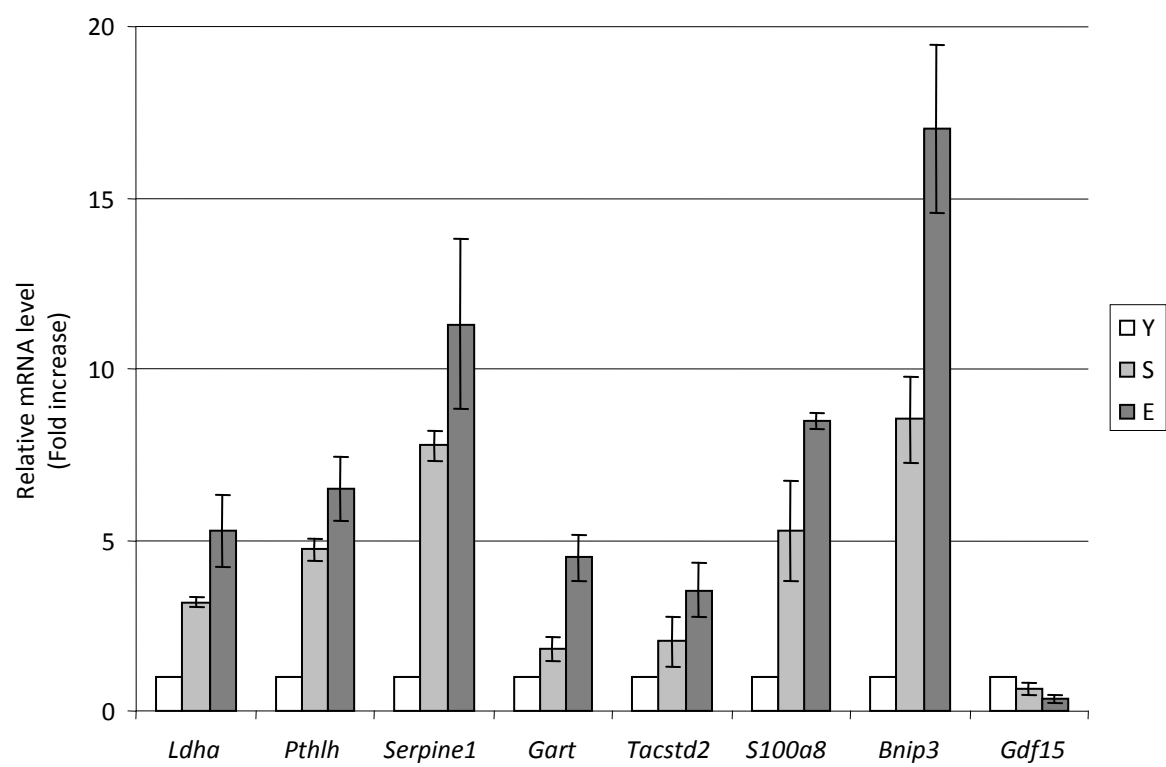

Figure S2

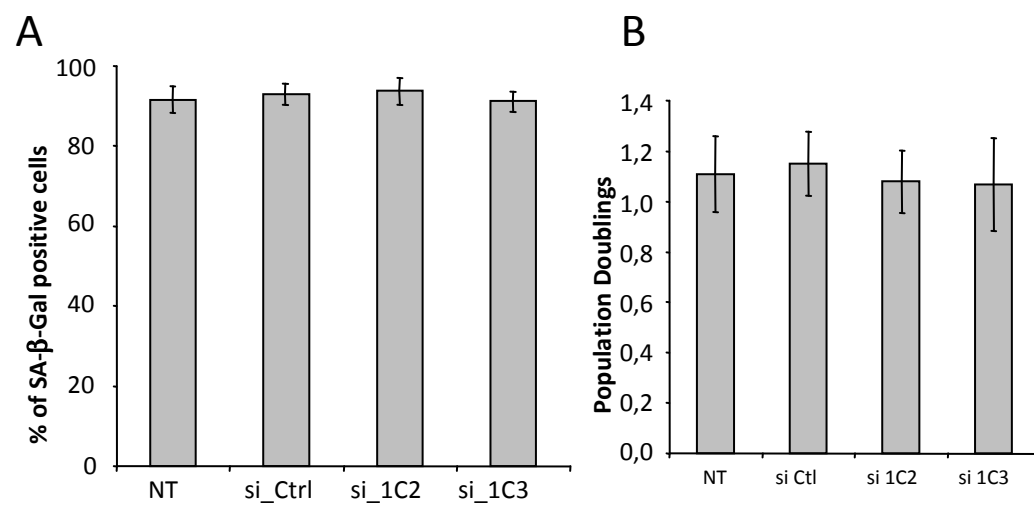

Figure S3

A

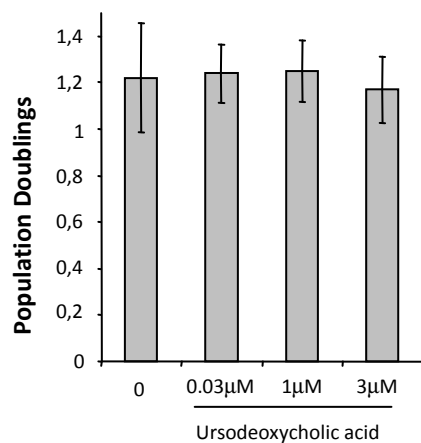

B

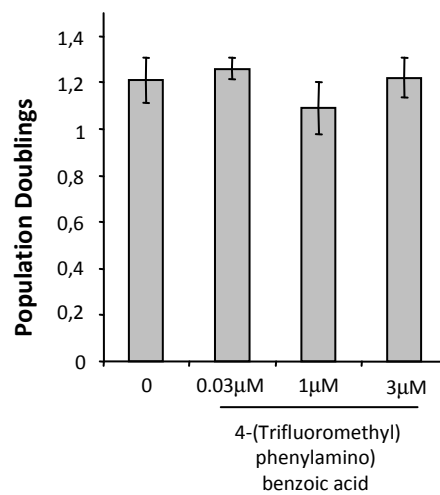

Figure S4

Supplement: Additional file 1: Figure S1 — Quality assessment of the microarray data. (A) MAPlots of log-ratio of two expression intensities versus the mean log-expression of the two upon loess normalization in the different conditions (E vs Y; E vs S). (B) Histograms of raw p-values representing the number of p-values that falls within different intervals. Figure S2. Validation of the microarray data by quantitative real-time PCR. The mRNA level of a random subset of genes were measured by qRT-PCR and normalized to 18S levels. The barplots represent the mean +/- s.d. of three independent experiments. Figure S3. AKR1C2 and 3 expressions do not alter the senescent state. Senescent NHEKs were subjected to AKR1C2 (si_1C2) and AKR1C3 (si_1C3) silencing by siRNAs or to non-target siRNAs (si_Ctrl) for 4 days, or were not transfected (NT). (A) SA-b-Gal-positive cells were counted in 3 different microscopic fields. The barplots represent the mean +/- s.d. of the 3 counts. (B) Cells were counted and cumulative doubling numbers were calculated. The barplots represent the mean +/- s.d. of the counts of three independent culture dishes. Figure S4. AKR1Cs inhibitors do not alter senescence growth arrest. (A and B) Senescent NHEKs were treated with ursodeoxycholic acid or 3-(4-2 (Trifluoromethyl) phenylamino) benzoic acid. After four days, cells were counted and cumulative doubling numbers were calculated. The barplots represent the mean +/- s.d. of the counts of three independent culture dishes. [file 1476-4598-13-151-S1.pdf]
