# Supplementary material for: Identification of a gene signature of a pre-transformation process by senescence evasion in normal human epidermal keratinocytes
Source: Mol Cancer. 2014 Jun 14;13:151. doi: 10.1186/1476-4598-13-151 (PMC4065601; doi:10.1186/1476-4598-13-151)
Supplement: Additional file 3: Table S2 — List of primers for qRT-PCR. [file 1476-4598-13-151-S3.pdf]

Table S2: List of primers for qRT-PCR.

Name	FWD	REV
AKR1C2	5’-GTTGTTTGAAAGTGTGTAGCA-3’	5’-GTCTTCACTTGGCTGGCA-3’
AKR1C3	5’-GGAGGGCTTTGCCTGATGT-3’	5’-GCTAAACAGGACGGATTTAAGT-3’
LDHA	5’-GGAGATCCATCATCTCTCCC-3’	5’-GGCCTGTGCCATCAGTATCT-3’
PTHLH	5’-TTGTCATGGAGGAGCTGATG-3’	5’-CGGTGTTCCTGCTGAGCTAC-3’
SERPINE1	5’-AGCTCCTTGTACAGATGCCG-3’	5’-ACAACAGGAGGAGAAACCCA-3’
GART	5’-TGCTGCAACCATGAGAAGAC-3’	5’-GCCCATATTACTGGTGGAGG-3’
TACSTD2	5’-TCCCGGGTTGTCATACAGAT-3’	5’-AATGTATCCCCTTTCGGTCC-3’
S100A8	5’-ACTTGTGGTAGACGTCGATGAT-3’	5’-CAGCTGTCTTTCAGAAGACCTG-3’
BNIP3	5’-CTGTGCGTCCAGCAGTATTT-3’	5’-GATGCAGGAGGAGAGCCTG-3’
GDF15	5’-AGAGATACGCAGGTGCAGGT-3’	5’-AGCTGGGAAGATTCGAACAC-3’
18S	5’-GTAACCCGTTGAACCCCATT-3’	5’-CCATCCAATCGGTAGTAGCG-3’
